# The Impact of a Very-Low-Calorie Ketogenic Diet in the Gut Microbiota Composition in Obesity

**DOI:** 10.3390/nu15122728

**Published:** 2023-06-13

**Authors:** Ana Karina Zambrano, Santiago Cadena-Ullauri, Patricia Guevara-Ramírez, Evelyn Frias-Toral, Viviana A. Ruiz-Pozo, Elius Paz-Cruz, Rafael Tamayo-Trujillo, Sebastián Chapela, Martha Montalván, Gerardo Sarno, Claudia V. Guerra, Daniel Simancas-Racines

**Affiliations:** 1 Centro de Investigación Genética y Genómica, Facultad de Ciencias de la Salud Eugenio Espejo, Universidad UTE, Quito 170527, Ecuador; santiagoa.cadena@ute.edu.ec (S.C.-U.); patyguevara28@gmail.com (P.G.-R.); viviana.ruiz@ute.edu.ec (V.A.R.-P.); elius.paz@ute.edu.ec (E.P.-C.); victor.tamayo@ute.edu.ec (R.T.-T.); 2 School of Medicine, Universidad Católica Santiago de Guayaquil, Guayaquil 090615, Ecuador; evelyn.frias@cu.ucsg.edu.ec; 3 Departamento de Bioquímica, Facultad de Ciencias Médicas, Universidad de Buenos Aires, Ciudad Autónoma de Buenos Aires C1121ABE, Argentina; sebachapela@gmail.com; 4 Hospital Británico de Buenos Aires, Equipo de Soporte Nutricional, Ciudad Autónoma de Buenos Aires C1280AEB, Argentina; 5 School of Medicine, Universidad Espíritu Santo, Samborondón 091952, Ecuador; mmontalvanmd53@gmail.com; 6 “San Giovanni di Dio e Ruggi D’Aragona” University Hospital, Scuola Medica Salernitana, 84131 Salerno, Italy; gsarno79@yahoo.it; 7 Centro de Investigación de Salud Pública y Epidemiología Clínica (CISPEC), Universidad UTE, Quito 170527, Ecuador; cguerra@ute.edu.ec (C.V.G.); dsimancas@ute.edu.ec (D.S.-R.)

**Keywords:** obesity, very-low-calorie ketogenic diet, nutrition, weight loss

## Abstract

The very-low-calorie KD (VLCKD) is characterized by a caloric intake of under 800 kcal/day divided into less than 50 g/day of carbohydrate (13%) and 1 to 1.5 g of protein/kg of body weight (44%) and 43% of fat. This low carbohydrate intake changes the energy source from glucose to ketone bodies. Moreover, clinical trials have consistently shown a beneficial effect of VLCKD in several diseases, such as heart failure, schizophrenia, multiple sclerosis, Parkinson’s, and obesity, among others. The gut microbiota has been associated with the metabolic conditions of a person and is regulated by diet interactions; furthermore, it has been shown that the microbiota has a role in body weight homeostasis by regulating metabolism, appetite, and energy. Currently, there is increasing evidence of an association between gut microbiota dysbiosis and the pathophysiology of obesity. In addition, the molecular pathways, the role of metabolites, and how microbiota modulation could be beneficial remain unclear, and more research is needed. The objective of the present article is to contribute with an overview of the impact that VLCKD has on the intestinal microbiota composition of individuals with obesity through a literature review describing the latest research regarding the topic and highlighting which bacteria phyla are associated with obesity and VLCKD.

## 1. Introduction

The ketogenic diet (KD) is a nutritional protocol characterized by a high fat and protein intake and low carbohydrate consumption. There are mainly four types of ketogenic diets: (1) classical KD, which is usually based on 90% fat, 4% carbohydrate, and 6% of proteins [1]. (2) medium-chain triglyceride, based on 10% long-chain triglycerides fat, 60% medium-chain triglycerides fat, 20% carbohydrate, and 10% protein [1]. (3) modified Atkins based on 65% fat, 10% carbohydrate, and 25% protein [1]. (4) low glycemic index diet based on 60% fat, 10% carbohydrate, and 30% protein [1].

The classical KD had suffered some variations; for instance, the low-calorie KD (LCKD) with a calorie intake of 800 to 1200 kcal/day based on 58% fat, 13% carbohydrate, and 29% of proteins, and the very-low-calorie KD (VLCKD) characterized by a caloric intake of under 800 kcal/day divided into less than 50 g/day of carbohydrate (13%) and 1 to 1.5 g of protein/kg of body weight (44%), and 43% of fat [2]. In these cases, the carbohydrate intake is reduced, forcing the body to switch to fatty acid oxidation, which induces ketogenesis. Ketogenesis is a metabolic pathway in which the triglycerides are hydrolyzed into fatty acids, and these into ketone bodies that could be used as alternative mitochondrial energy [3,4,5]. VLCKD, fasting, and exercise promote glucose consumption, lowering the insulin level and converting fatty acids into ketone bodies [3,6]. During ketogenesis, there are three types of ketone bodies, acetone, acetoacetate (AcAc), and the mainly produced 3-hydroxybutyrate (BHB) [3]. The ketone bodies are broken down in the mitochondria of metabolically active cells into acetyl-CoA, then to ATP [3,4,6].

There are reports with clinical trials of the metabolic effect of ketone bodies from the VLCKD implicated in the prevention and treatment of human diseases. Clinical trials have consistently shown a beneficial effect of VLCKD in improving heart failure [7,8], neuroprotective properties in schizophrenia, multiple sclerosis, Parkinson’s, and Alzheimer’s diseases [9,10,11,12], improving inflammation [13], reducing obesity [2,14,15,16,17], and recovering muscle force after critical illness [18,19], among others.

Obesity has been described as the accumulation of fat in an individual, causing a direct impact on a person’s health and daily life [20]. Moreover, the World Health Organization (WHO) declared obesity a global epidemic when the individual’s body mass index (BMI) is equal to or greater than 30 kg/m^2^ [20,21]. Approximately 13% of the adult population was obese in 2016, and it is expected that almost the majority of the population will be obese in 2030 [21,22]. There are reports of the adverse health impact or co-morbidities promoted by obesity, such as heart diseases, hypercholesterolemia, hypertension, and depression [23,24,25,26,27,28]. Additionally, obesity may cause endothelial, inflammatory, and hormonal alterations, which may lead to a hypertensive state, increasing cardiovascular disease (CVD) predisposition [29]. There is increased concern regarding CVD and its correlation with obesity due to epidemiological data, which have shown a direct linear relationship between both; hence if the prevalence of obesity increases, the CVD prevalence will also increase [30]. If there is no adequate follow-up and management of people with obesity, the adverse effects can lead to serious complications and could cause the person’s death [20]. Therefore, it is important to elucidate the etiology of obesity to develop effective interventions to diminish the burden of the disease.

In the last few years, the keto diet has gained popularity as an alternative to reducing weight by lowering appetite through the production of ketone bodies [31]. However, within the diets, a study by Bezerra Bueno N. et al. (2013) [32] compared the conventional low-fat diet in the long term and the VLCKD; the authors found that VLCKD led to a more substantial decrease in weight, making it an excellent alternative for weight loss [32].

Furthermore, the gut microbiota is regulated by diet interactions and has been correlated with the metabolic conditions of a person [33]. Hence, the microbiota composition is variable depending on the environment and dietary patterns of the subjects. For instance, research has shown that the human virome could be correlated to disease development, principally due to its influence on the microbiota [34]. This gut microbiota variability between individuals may make it difficult to identify differences in the composition when comparing diets in people with obesity [35,36]. However, the essential role of the microbiome in the body weight homeostasis maintenance, regulating metabolism, appetite, and energy, has been demonstrated by producing compounds derived from bacteria and influencing the metabolic pathways of the individual [37,38].

The objective of the present article is to provide an overview of the impact that VLCKD has on the intestinal microbiota composition of individuals with obesity through a literature review describing the latest research regarding the topic and highlighting which bacteria are associated with obesity and VLCKD.

## 2. Gut Microbiota in Obesity

There are reports that changes in the gut microbiota composition of individuals with obesity compared to healthy individuals are not a consequence of obesity. For instance, Turnbaugh P. et al. (2008) [39] showed that colonization of germ-free mice with microbiota associated with obesity led to an increase in fat deposition and total body fat. Hence, the gut microbiota is a contributing factor to obesity pathophysiology [39,40].

The Human Microbiome Project has reported that healthy gut microbiota is mainly composed of Firmicutes, Bacteroidetes, Actinomycetes, Proteus, Fusobacteria, and Verrucomicrobia [41]. Based on these findings, several studies have compared the differences in the microbial community in healthy and individuals with obesity. The first reports are from observational studies performed on animal models. For instance, Ley R.E. et al. (2006) [41] showed fewer Bacteroidetes and more Firmicutes abundance compared to their not obese mice controls. Moreover, the coexistence of both microorganisms minimized the competition for resources, but uncharacterized properties increased the Firmicutes proportion [42]. Similarly, another study by Turnbaugh P. et al. (2006) [40] demonstrated in animal models that the microbiota associated with obesity increases the ability to obtain energy from the diet, and the increase in the Firmicutes/Bacteroidetes ratio in the microbiota was associated with an obese phenotype [39].

Bifidobacterium species are an important and beneficial compound of gut microbiota; they may be used as probiotics due to their role in producing acetate and lactate after glucose fermentation. For instance, Waldram A. et al. (2009) [43] performed a study using spectroscopy, fluorescence in situ hybridization (FISH), and electrophoresis to identify the structural differences between obese and lean mice. The authors identified a reduction in the abundance of Bifidobacterium compared to healthy individuals, suggesting a possible inverse association between Bifidobacterium and obesity [43].

Similar dysbiosis has been described to occur in humans, Duan M. et al. (2021) [44] showed significant differences in the gut microbiota between a control group and adults with obesity. The authors found a clear reduction in the gut microbiota diversity of the latter. The results in the obesity group, at the phylum level, exhibited a decrease in Firmicutes, an increase in Bacteroidetes, and a reduction in the Firmicutes/Bacteroidetes ratio. Similarly, Actinobacteria and Fusobacteria had significantly different proportions. At the species level, nine species, including Fusobacterium mortiferum, Faecalibacterium prausnitzii, Bacteroides uniformis, and Barnesiella intestinihominis, were markedly distinct. Moreover, the lipid and carbohydrate metabolism pathways were abnormal. According to the authors, the changes in Firmicutes and Bacteroidetes could be related to the environmental conditions of the individuals, as the diet of the obese group under study preferred food made of flour [44].

Likewise, Schwiertz A. et al. (2010) [45] performed a study to evaluate the differences in gut microbiota and short-chain fatty acid (SCFA) concentration between adults with obesity and lean subjects in Germany. Regarding the SCFA concentration, the authors found a higher concentration in the obesity group in comparison with the control group. They also reported smaller proportions of Firmicutes and an increased abundance of Bacteroidetes in adults with obesity in contrast with other reports, for instance, the previously mentioned study by Duan M. et al. (2021) [44]. Furthermore, bacteria from the Euryarchaeota phylum (Methanobrevibacter) and Actinobacteria (Bifidobacterium) were detected in lower concentration proportions in the obesity group. Bacteria from Firmicutes and Bacteroidetes produce SCFA, mainly butyrate and propionate, which may perform an important role in obesity. Additionally, SCFA can avoid digestion in the small intestine and be an additional energy source [45].

On the other hand, Koliada A. et al. (2017) [46] carried out a study to understand the association between the gut microbiota, with a special focus on the Firmicutes/Bacteroidetes ratio and BMI in a sample from the Ukranian population. The authors compared the fecal concentrations of the Actinobacteria, Firmicutes, and Bacteroidetes phyla. The results showed differences in the proportion of each bacterial group between the groups. The abundance of Firmicutes increased with a higher BMI, whereas the proportion of Bacteroidetes decreased with an increasing BMI. The Firmicutes/Bacteroidetes ratio also grew with a higher BMI. These results may be explained by the fact that Firmicutes are better energy sources than Bacteroidetes, leading to higher calorie absorption and subsequent weight gain [46].

Similarly, Zhang H. et al. (2009) [47] studied the gut microbiota in people with obesity, after a gastric bypass, and in normal-weight adults. The phylogenetic analyses showed that the Firmicutes phylum was highly abundant in the normal-weight and obese groups; however, after a gastric bypass, the abundance markedly decreased. Moreover, there was a significant increase in the Prevotellaceae family, mainly associated with H2 production, in adults with obesity. The findings led to the hypothesis that the interspecies H2 transfer is an important mechanism for increasing energy uptake in individuals with obesity. Furthermore, the results also indicate the impact that a surgical procedure may have on microbiota [47].

A study by Bervoets L. et al. (2013) [48] identified the differences in the gut microbiota composition between obese and lean children. The authors found that children with obesity had a higher Firmicutes/Bacteroidetes ratio and a higher concentration of Lactobacillus spp. (Firmicutes phylum) [48]. Interestingly, the Lactobacillus genus has been positively and negatively associated with obesity. For instance, Million M. et al. (2013) [49] showed that Lactobacillus reuteri was correlated with a higher BMI [49], whereas Karlsson F.H. et al. (2013) [50] found that Lactobacillus casei was negatively related to obesity [50]. These results suggest a possible strain-specific role of the Lactobacillus genus in obesity [48,49,51].

Xu Z. et al. (2022) [52] performed a systematic review correlating microbiota, obesity, and metabolic disorders, analyzing 2390 reports and including 60 studies. The authors found that Proteobacteria was the most associated phylum with obesity, followed by Firmicutes. Furthermore, the authors also described Bacteroidetes and Actinobacteria as lean-associated phyla [52]; for instance, Bai J. Hu Y. Bruner DW. (2019) found that an increase in the Proteobacteria phylum was correlated with a higher BMI in a cohort of 7–18 years old children [53].

Finally, Moreno-Navarrete J.M. et al. (2018) [54] analyzed the correlation between gut microbiota, insulin sensitivity, and gene expression in subcutaneous and visceral tissue in subjects with obesity. The authors found that the individuals with insulin resistance had an increased abundance of Bacteroidetes and Proteobacteria; and a decrease in the Firmicutes proportion. They also identified that the relative abundance of Firmicutes was associated with markers of brown adipocytes in subcutaneous obesity but not visceral obesity [54].

Obesity has not been associated with a specific bacteria or pathogen, but it may be directly related to dysbiosis in the ecosystem, which is directly influenced by diet and the environment in which the individual develops. For this reason, differences in the abundance of several bacterial strains have been identified in models and patients with obesity.

## 3. Impact of a Very-Low-Calorie Ketogenic Diet (VLCKD) on the Microbiota of Subjects with Obesity

VLCKD involves carbohydrate deprivation, which is associated with a decrease in glycolysis and an increase in lipolysis, glycogenolysis, and gluconeogenesis for energy generation [55]. During lipolysis, ketone bodies (acetone, 3-β-hydroxybutyrate, acetoacetate) are generated and used as an energy source [55]. Interestingly, ketone bodies can produce more energy than glucose because of the ketosis metabolic effects [36]. KD has also been related to a decrease in the synthesis of reactive oxygen species and the upregulation of energy metabolism genes, mitochondrial biogenesis, and K_ATP_ channels [55]. Research has analyzed the impact that VLCKD has on obesity. For instance, Barrea L. et al. (2023) [30] performed a study on 137 women that agreed to participate in the clinical trial. The authors found that after 45 days of VLCKD, all the women in the project experienced a significant weight reduction and improvement in body composition parameters. Furthermore, research regarding VLCKD, and microbiota modulation is lacking [30].

Gutiérrez-Repiso C. et al. (2019) [56] performed a nutritional intervention clinical trial to determine the effects of a VLCKD and symbiotic on the microbiota of thirty-three adults with obesity [56]. The authors found an association between VLCKD and weight loss. They also reported a significant difference in the gut microbiota diversity by analyzing the Shannon Index, which is a measure of species diversity at distinct levels [56,57]. At the genus level, *Butyricimonas* and *Oscillospira* abundance increased. *Oscillospira*, belonging to the *Firmicutes* phylum, has been positively associated with high-density lipoprotein, butyrate, leanness, human health, and microbial diversity [58,59], whereas the *Butyricimonas* genus (*Bacteroidetes* phylum) has been positively correlated to energy metabolism, promoting the homeostasis between microbiota and the host [60,61]. On the other hand, the proportion of *Erwinia*, *Serratia*, and *Citrobacter* decreased. Interestingly, an increased abundance of *Serratia* and *Citrobacter* has been associated with obesity [62,63]. The authors concluded that VLCKD could restore the microbiota after obesity-associated dysbiosis [56].

Additionally, in another study by Gutierrez-Repiso A. et al. (2021) [64], the authors compared the microbiota of subjects with different weight loss interventions (Mediterranean diet, VLCKD, and sleeve gastrectomy bariatric surgery) [64]. They found that VLCKD patients had an increased abundance of *Alistipes* and *Parabacteroides*. Correlations between the decreased proportion of *Alistipes* and *Parabacteroides* and obesity have been described [44,65]. On the contrary, the authors found a decrease in *Lactobacillus* [64]. The effect of the *Lactobacillus* genus on obesity appears to be strain dependent. For instance, studies have associated decreased visceral fat, BMI, and waist circumference with an increased abundance of *L. gasseri* [66]. Similarly, Wang M. et al. (2020) [67] described a correlation between the presence of *L. fermentum*, *L. acidophilus*, *L. casei*, *L. paracasei*, and *L. rhamnosus* and a decrease in body weight [67], whereas strains, such as *L. reuteri,* have been positively associated with obesity [68].

Basciani S. et al. (2020) [69] analyzed the effects of VLCKD on body composition parameters and gut microbiota of subjects with obesity. The authors enrolled forty-eight subjects and divided them into three groups: (1) VLCKD with whey protein, (2) VLCKD with vegetable protein, and (3) VLCKD with animal protein [69]. All groups showed a significant reduction in total fat mass, body weight, total and low-density lipoprotein cholesterol, BMI, triglycerides, and waist, thigh, and hip circumference. Moreover, at baseline, they found a higher abundance of *Firmicutes*, followed by *Bacteroidetes*, *Proteobacteria*, and *Actinobacteria*. However, at day 45 of the VLCKD, the authors found a decreased proportion of *Firmicutes* and *Actinobacteria* and an increased abundance of *Bacteroidetes* and *Proteobacteria* [69]. Alterations in the *Firmicutes/Bacteroidetes* ratio have been broadly associated with several diseases, including obesity [44,70]. For instance, Palmas V. et al. (2021) [65] described a positive association between obesity and an increased *Firmicutes/Bacteroidetes* ratio, reporting values more than twice in comparison with the microbiota of subjects with standard weight [65]. Similarly, an increased abundance of the *Actinobacteria* phylum has been correlated with obesity [71,72]. Moreover, the *Proteobacteria* phylum has been related to obesity as a potential inflammation driver [52]. Additionally, the authors found that the whey and vegetable protein groups showed the highest decrease in *Firmicutes* abundance, whereas the whey protein group had the highest increase in *Bacteroidetes* proportion [69].

Likewise, Deledda A. et al. (2022) [73] analyzed the dynamics of the gut microbiota by comparing the effect of a Mediterranean diet (MD) and a VLCKD [73]. The authors described that both diets influenced weight reduction, BMI, and waist circumference, and identified an increased abundance of the *Verrucomicrobiota* phylum, and characterized the *Akkermansiaceae* and *Christensenellaceae* families as microbial markers associated with VLCKD [73]. Interestingly, members of the *Akkermansiaceae* family as the *A. municiphila* have been correlated with anti-obesogenic effects in rodents and humans [74,75,76]. Even though the *Christensenellales* family belongs to the *Firmicutes* phylum, its abundance has been described as enriched in individuals with normal BMI in comparison with subjects with obesity [77]. The authors also described a depletion in the abundance of the *Actinobacteria* phylum, which has been associated with obesity [71,72]. In addition, the strongest gut microbiota positively associated pathways included non-homologous end-joining pathways and steroid and carotenoid biosynthesis. On the other hand, cephalosporin, penicillin biosynthesis, pinene, limonene, and ethylbenzene degradation were negatively associated with VLCKD. No significant taxa or pathway differences were found for the MD group [73].

**Figure 1:**
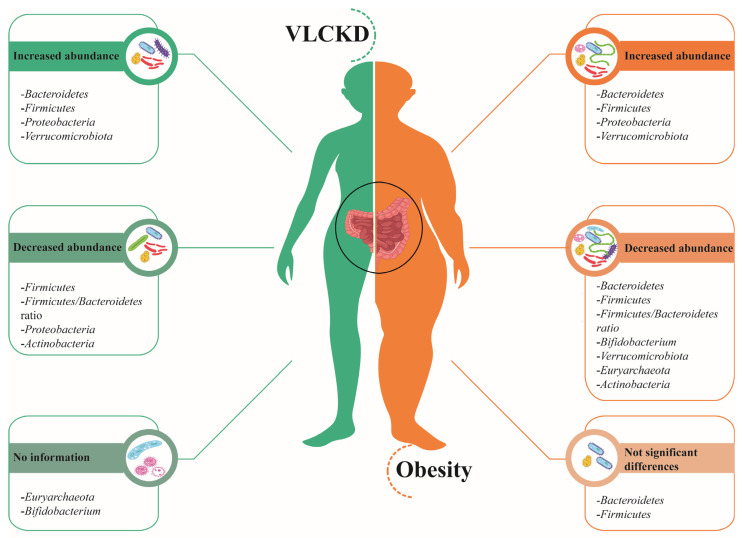
Gut microbiota in VLCKD and obesity. On the left side (green), bacteria phyla with an increased or decreased abundance after a VLCKD are represented, whereas on the right side (orange), the phyla associated with obesity are depicted [42,43,44,45,46,55,63,68,72].

The VLCKD Is an excellent alternative for weight loss on obesity management. Additionally, research has shown how VLCKD could improve microbiota homeostasis, promoting the abundance of bacteria associated with good health. Figure 1 and Supplementary Table S1 compare the reported effects that obesity and VLCKD have on gut microbiome abundance.

## 4. Discussion

Researchers consider the gut microbiota a “hidden metabolic organ” because of its role in host brain function, immunity, inflammation, nutrition, and metabolism [78,79]. Estimates suggest that there are around 500 to 1000 bacterial species at any given time in our gut, with each strain having a unique genome [80,81]. The bacteria in the microbiota produce an extensive range of metabolites, such as short-chain fatty acids (SCFAs), serotonin, and nitric oxide, which, based on their chemical structure similarity, can bind to host cell receptors and trigger hormonal signaling. For instance, it has been shown that strains of Lactobacilli can produce γ amino butyric acid (GABA), an inhibitory neurotransmitter whose modulation can influence depression and anxiety [82,83].

The human gut microbiota is mainly composed of *Firmicutes* and *Bacteroidetes*, which are the most abundant, followed by *Proteobacteria* and *Actinobacteria* phyla. Moreover, the principal enterotypes include *Ruminococcus*, *Bacteroides*, and *Prevotella* [84]. Interestingly, it has been shown that the host’s diet determines which genus is the most abundant [85]. Furthermore, microbiome dysbiosis has been correlated with several diseases, including epilepsy, Alzheimer, inflammatory bowel syndrome, and obesity [55,69]. Cuevas-Sierra A. et al. (2019) [86] reported a significant difference in gut microbiota between lean and subjects with obesity [86]. The gut microbiota is influenced by various factors, such as antibiotic use, genetics, age, and diet [33,36,73,80]. Additionally, this microbiota modulation could potentially help to treat these diseases [69].

Obesity has been associated with various metabolic diseases as hypertension and diabetes, among others. Several factors promote the pathogenicity of obesity as an energy imbalance, low-grade inflammation, and gut microbiota dysbiosis [87,88,89]. For instance, an increased *Firmicutes/Bacteroidetes* ratio is considered one of the hallmarks of obesity, although this statement has been disputed. Moreover, it has been shown that an altered microbiota increases gut permeability, which could lead to the passage of endotoxins, such as lipopolysaccharides (LPS). LPS may trigger a constant inflammatory and immune response [73,84,90]. In addition, Meijnikman AS. et al. (2020) [91] found that subjects with a higher BMI had a lower gut microbiota alpha diversity compared to non-obese individuals [91]. The authors also described a positive correlation between gut microbial amino acid metabolism and obesity [91].

The impact of diet on the microbial population has been well described; however, recent studies have highlighted that the microbiome may also influence nutritional status via their secreted metabolites, such as SCFAs, secondary bile acids (Bas), and indole metabolites, which could alter appetite regulation by modifying the activity of the enteric nervous system and the gut-brain axis, generating bi-directional crosstalk [84,92]. Enteroendocrine cells are located in the stomach, pancreas, and gut epithelium, and they can respond to nutrients, metabolites, and mechanical stimuli by secreting neurotransmitters, such as glucagon-like peptide 1, ghrelin, serotonin, and peptide YY. These neurotransmitters are associated with several essential functions in metabolism and can modulate gut motility, insulin and bile acids secretion, and food intake [84,92]. For instance, Modasia A. et al. (2020) [93] showed that the gut bacterium *Bacteroides thetaiotaomicron* could influence enteroendocrine cells in the gastrointestinal tract of a murine model [93].

Studies have compared the impact of different diets on the microbiome. For instance, as previously mentioned, Deledda A. et al. (2020) [73] compared the effects of a VLCKD and an MD. The authors found that, in addition to a reduction in body weight and BMI, the VLCKD had a statistically significant impact on gut microbiota, promoting the abundance of leanness-associated bacterium, such as *A. municiphila* [73]. Similarly, MD is based on the reduced consumption of red meat, and unprocessed and industrial foods may influence body weight and BMI. However, the effect on the microbiota before and after the nutritional intervention did not achieve statistically significant differences [73].

Likewise, Simões CD. et al. (2013) [94] analyzed the effects of a very low-energy diet (VLED) on gut microbiota. The authors found a two-fold decrease in *Bifidobacterium* after the nutritional intervention [94]. Interestingly, although *Bifidobacterium* belongs to the *Actinobacteria* phylum, which has been positively correlated to obesity, this genus is considered a probiotic associated with leanness and good health [94,95]. Moreover, the study also showed that the changes in gut microbiota were promoted by diet and not due to weight changes [94]. In comparison with VLCKD, the VLED changes in gut microbiota were only significant for one genus, whereas, for the VLCKD, the changes were significant in the increased or decreased abundance of different phyla. However, the approaches to evaluate the microbiome were different due to Simões CD. et al. 2014 used qPCR and FISH methods for VLED, and the VLCKD analyses mentioned in this paper were performed using next-generation sequencing. More research is needed to understand the differences in gut microbiota modulation between a VLED and a VLCKD.

Furthermore, Remely M. et al. (2015) [96] analyzed the effect of intermittent fasting (IF) on the microbiota. The authors did not report significant changes in total bacteria abundance; however, they found an increased proportion of the *Faecalibacterium prausnitzii* species and the *Akkermansia* and *Bifidobacterium* genera [96]. A decline in *Faecalibacterium prausnitzii* abundance has been associated with chronic inflammation and obesity [97]. Similarly, a reduction in the *Akkermansia* and *Bifidobacterium* genera has been correlated with obesity in different studies [95,98]. Similar to VLCKD, the influence of IF on gut microbiota looks promising; however, further studies should be conducted to identify the role of IF on the microbiome.

Miao Z. et al. (2022) [99] studied the effect of a plant-based diet on gut microbiota. The authors found an increased alpha and beta diversity after a healthy plant-based diet [99]. Similarly, Losasso C. et al. (2018) [100] compared the gut microbiota of three groups (vegetarian, vegan, and omnivore). The group found an increased microbiome diversity, increased *Bacteroidetes* abundance, and a decreased *Firmicutes* proportion. Although disputed, an increased microbiota diversity has been positively associated with greater functional diversity and host health [100]. In comparison with the VLCKD, a plant-based diet appears to have a stronger influence on the *Firmicutes/Bacteroidetes* ratio. More studies evaluating plant-based and VLCKDs must be conducted to associate the effect of diet on the microbiome.

## 5. Conclusions

There are several factors associated with the etiology of obesity, primarily associated with genetics, diet, and lifestyle. The prevalence of obesity is increasing and is estimated to affect half of the population in the future, which is immensely alarming. There is increasing evidence of an association between some bacterial strains and weight. VLCKD is a remarkable option for weight loss because, due to carbohydrate deprivation, triglycerides, and fatty acids are catabolized into ketone bodies, which are a more efficient energy source than glucose. Furthermore, besides the impact that VLCKD has on weight loss, it has been shown that VLCKD could significantly modulate gut microbiota and restore its homeostasis, which has been an essential and integral part of the treatment for several diseases, such as epilepsy, Alzheimer, inflammatory bowel syndrome, and obesity, as mentioned in the review. Additionally, by comparing the VLCKD with other types of diet, only the plant-based diets had a similar beneficial effect on gut microbiota, highlighting the impact that a VLCKD has on the microbiota.

## 6. Future Directions

Currently, there is increasing evidence of an association between gut microbiota dysbiosis and the pathophysiology of obesity. In addition, the molecular pathways, the role of metabolites, the interactions with viruses, and how microbiota modulation could be beneficial remain unclear, and more research is needed. Developments in the metagenomics field have allowed us to characterize each person’s microbiome, the metabolites produced, and their related pathways. This new information is vital to understand the association between microbiota and host health by identifying new disease biomarkers and how they can be modulated to promote homeostasis. However, there is a discrepancy regarding the “good” microbiota. For instance, some studies associate a decrease in the abundance of Firmicutes with a reduction in BMI, whereas others have related this effect to a drop in the Bacteroidetes proportion. Hence, it is highly important to elucidate which bacterial strains are beneficial for our health.

## Data Availability

The results are presented in the paper. For more information, please contact the corresponding author.

## Supplementary Materials

The following supporting information can be downloaded at: https://www.mdpi.com/article/10.3390/nu15122728/s1, Table S1: Gut microbiota microorganisms’ abundance in individuals with obesity compared to individuals on a VLCKD.

## Abbreviations

Very low-calorie ketogenic diet	VLCKD
Ketogenic diet	KD
Low-calorie ketogenic diet	LCKD
Acetoacetate	AcAc
3-hydroxybutyrate	BHB
World Health Organization	WHO
Body mass index	BMI
Cardiovascular disease	CVD
Short-chain fatty acids	SCFA
Mediterranean diet	MD
Gamma amino butyric acid	GABA
Lipopolysaccharides	LPS
Very low-energy diet	VLED
Intermittent fasting	IF
