# Supplementary material for: The Impact of a Very-Low-Calorie Ketogenic Diet in the Gut Microbiota Composition in Obesity
Source: Nutrients. 2023 Jun 13;15(12):2728. doi: 10.3390/nu15122728 (PMC10305724; doi:10.3390/nu15122728)
Supplement: Supplementary file 1 [file nutrients-15-02728-s001.zip › nutrients-2394935-supplementary.pdf]

Supplementary Table S1.- Gut microbiota microorganisms' abundance in individuals with obesity compared to individuals on a VLCKD.

<table>
<tr><th>Microorganism</th><th>Abundance in obesity</th><th>Abundance in VLCKD diet</th></tr>
<tr><td rowspan="3">Bacteroidetes</td><td>Decreased abundance [41,45]</td><td rowspan="3">Increased abundance [55,63,68]</td></tr>
<tr><td>Increased abundance [43,44]</td></tr>
<tr><td>Not significant differences [46]</td></tr>
<tr><td rowspan="4">Firmicutes</td><td rowspan="2">Increased abundance [41,45,47,48,50]</td><td>Increased abundance [55,72]</td></tr>
<tr><td rowspan="3">Decreased abundance [63,68]</td></tr>
<tr><td>Decreased abundance [43,44]</td></tr>
<tr><td>Not significant differences [46]</td></tr>
<tr><td rowspan="2">Firmicutes/Bacteroidetes ratio</td><td>Increased ratio [38,45,47]</td><td rowspan="2">Decreased ratio [68]</td></tr>
<tr><td>Decreased ratio [43]</td></tr>
<tr><td rowspan="2">Proteobacteria</td><td rowspan="2">Increased abundance [52]</td><td>Increased abundance [68]</td></tr>
<tr><td>Decreased abundance [55]</td></tr>
<tr><td>Verrucomicrobiota</td><td>Decreased abundance [72]</td><td>Increased abundance [72]</td></tr>
<tr><td>Bifidobacterium</td><td>Decrease abundance [42,44]</td><td>No information</td></tr>
<tr><td>Euryarchaeota</td><td>Decrease abundance [44]</td><td>No information</td></tr>
<tr><td rowspan="2">Actinobacteria</td><td>Decrease abundance [42,44]</td><td rowspan="2">Decreased abundance [68,72]</td></tr>
<tr><td>Increase abundance [45]</td></tr>
</table>
